# Characterization and Purification of Bergamottin from *Citrus grandis* (L.) Osbeck cv. Yongjiazaoxiangyou and Its Antiproliferative Activity and Effect on Glucose Consumption in HepG2 cells

**DOI:** 10.3390/molecules22071227

**Published:** 2017-07-20

**Authors:** Yilong Liu, Chuanhong Ren, Yunlin Cao, Yue Wang, Wenyi Duan, Linfeng Xie, Chongde Sun, Xian Li

**Affiliations:** 1 Zhejiang Provincial Key Laboratory of Horticultural Plant Integrative Biology, Zhejiang University, Zijingang Campus, Hangzhou 310058, China; yilongliu@zju.edu.cn (Y.L.); 21616051@zju.edu.cn (C.R.); 21516059@zju.edu.cn (Y.C.); fruit@zju.edu.cn (Y.W.); duanwenyi@zju.edu.cn (W.D.); linfengxie@zju.edu.cn (L.X.); adesun2006@zju.edu.cn (C.S.); 2 Laboratory of Fruit Quality Biology, Zhejiang University, Zijingang Campus, Hangzhou 310058, China

**Keywords:** bergamottin, purification, column chromatography, HSCCC, antiproliferative activity, glucose consumption

## Abstract

Bergamottin is a natural furanocoumarin compound with weak polarity. Characterization and quantification of bergamottin were carried out in different fruit tissues of various citrus cultivars. Among the four citrus tissues tested, i.e., flavedo, albedo, segment membrane (SM), and juice sacs (JS) in eight citrus cultivars, the highest bergamottin content was found in the flavedo of *Citrus grandis* (L.) Osbeck cv. Yongjiazaoxiangyou (YJZXY, 666.54 μg·g^−1^ DW). A combination of silica gel column chromatography and high-speed counter-current chromatography (HSCCC) was established to efficiently purify bergamottin from the flavedo of YJZXY. Bergamottin showed significant antiproliferative activity on three cancer cell lines, i.e., human liver cancer HepG2, promyelocytic leukemia HL-60, and gastric cancer BGC-823 cells, which showed a marked inhibition effect on these cell lines in a dose-dependent manner. In addition, bergamottin significantly increased glucose consumption in HepG2 cells also in a dose-dependent manner, which is the first report of its potential in anti-diabetes applications.

## 1. Introduction

**Figure 1:**
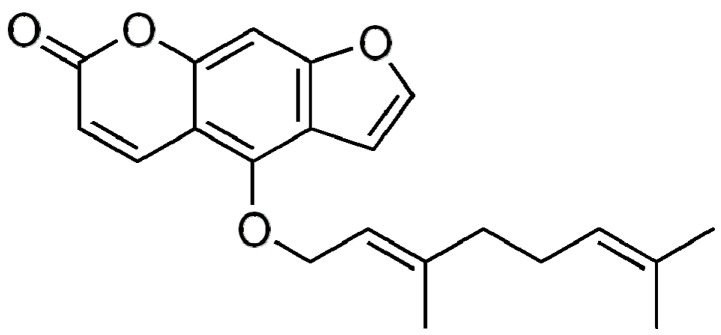
Structure of bergamottin.

Bergamottin, i.e., 5-geranoxypsoralen, is a natural furanocoumarin compound with weak polarity (Figure 1). Recent studies showed that bergamottin contains various pharmaceutical bioactivities, including anticancer (anti-proliferation, anti-invasion, and anti-migration, etc.) [1,2,3], antimycobacterial activity [4], antimutagenicity [5], and increased drug bioavailability through its interaction with some isoforms of the cytochrome P450 enzyme [6,7,8], etc. However, such investigations on bergamottin bioactivities are limited in in vitro cell studies due to the rarity and expensiveness of this compound. Few studies investigated further the bioavailability and metabolism of bergamottin in vivo. Therefore, characterization of the distribution of bergamottin in natural resources and establishment of well-defined separation methods for efficient purification of this natural bioactive compound will facilitate its further comprehensive utilization.

Bergamottin was originally detected in the oil of bergamot (*Citrus bergamia*) as early as 1937 [9]. Recently, the biological activities of the essential oil of bergamot have been widely studied [10,11,12] and bergamottin has attracted much attention as the major component [13]. Besides the essential oil of bergamot, bergamottin was also reported in the fruits of the *Citrus* family such as in lemon oil [14], grapefruit juice [15,16,17], the juice sac and segment epidermis of bergamot [18], the peel of *Citrus aurantifolia* [4], etc. In addition, bergamottin was also reported in some traditional Chinese medicinal herbs such as the roots and rhizomes of *Notopterygium incisum* [19]. The distribution of bergamottin in plant resources was found to vary with plant species, variety, tissues, location and stage of maturity, etc. In one study comparing the content of four furanocoumarin monomers in different citrus germplasms, all the tested grapefruits and the majority of pomelos were found to contain higher bergamottin content than other citrus cultivars such as mandarins, lemons and sweet oranges [17]. Furthermore, such content of bergamottin in different citrus cultivars varied with the locations of the cultivation and different years [17], etc. So far, no further study has compared the bergamottin content in different fruit tissues, i.e., flavedo, albedo, segment membrane (SM), juice sac (JS) of different citrus cultivars at the same time.

Silica gel column chromatography separated the components in the mixture according to their different adsorption force on silica gel. Silica gel exhibits adsorption properties by forming hydrogen bonds through the hydroxyl groups of silicon atoms with compounds. In general, compounds with higher polarities are easily adsorbed by the resin while compounds with lower polarities will be easily separated by elution solutions. Silica gel column chromatography is easy to operate and has been successfully applied for the separation of compounds with low polarity [4,20]. In the past decade, high-speed counter-current chromatography (HSCCC) has shown distinguished purification characteristics based on its liquid–liquid partition mechanism. By using liquid as the stationary phase, HSCCC has less or no irreversible adsorption, low risk of sample denaturation, high recovery and low cost, the advantages of which enable its application for the purification of important natural compounds with quite satisfactory yield [21,22]. Therefore, combination of silica gel column chromatography and HSCCC would be an efficient and rapid technique to isolate a number of natural products with low polarity from natural resources.

The present study was designed to investigate the distribution of bergamottin in different fruit tissues of different citrus resources and to establish an efficient purification procedure to isolate bergamottin with high purity and yield by using the combination of silica gel column chromatography and HSCCC. The antiproliferative activity of bergamottin against three cancer cell lines, i.e., human liver cancer HepG2 cells, promyelocytic leukemia HL-60 cells, and gastric cancer BGC-823 cells, was then studied. Furthermore, the effects of bergamottin on glucose consumption in HepG2 cells were also investigated for its possible role in antidiabetic activity through regulating the glucose metabolism in vitro.

## 2. Results and Discussion

### 2.1. Quantification of Bergamottin in Different Fruit Tissues of Eight Citrus Cultivars

In order to investigate the distribution of bergamottin in different fruit tissues, citrus fruit were separated into four parts, i.e., flavedo, albedo, SM and JS. Results showed that the bergamottin content was significantly different among the four tissues, and flavedo showed the highest bergamottin content in all the cultivars tested (Table 1). Therefore, citrus flavedo is a good source of bergamottin. In addition, there was significant difference in the bergamottin content among the eight citrus cultivars tested. The flavedo of Yongjiazaoxiangyou (YJZXY) showed the highest bergamottin content (666.54 μg·g^−1^ DW) and therefore it was chosen as the purification material for further study. 

As the edible part of citrus fruit, JS of some cultivars such as red grapefruit also showed relatively high bergamottin content (144.24 μg·g^−1^ DW) (Table 1). Bergamottin has been reported as one of the principal components in grapefruit juice in several studies [6,8,23]. Since bergamottin content in fruit is affected by both genetic and environmental factors, factors such as cultivar, harvest season and year, storage time, production site, pre- and post-harvest treatment should be taken into consideration in evaluating different plant materials [17,24,25].

### 2.2. Purification of Bergamottin from the Flavedo of YJZXY

Since the flavedo of YJZXY was used as the material to purify the low-polarity bergamottin, removal of the hydrophobic pigment and essential oil in the flavedo was the main challenge. The use of silica gel column chromatography resulted in the removal of the majority of impurities where petroleum ether/ethyl acetate 13:1 (*v*/*v*) was selected as the elution solvent. The pooled eluent was then evaporated in vacuum as the silica gel-refined sample for HSCCC purification. 

**Figure 2:**
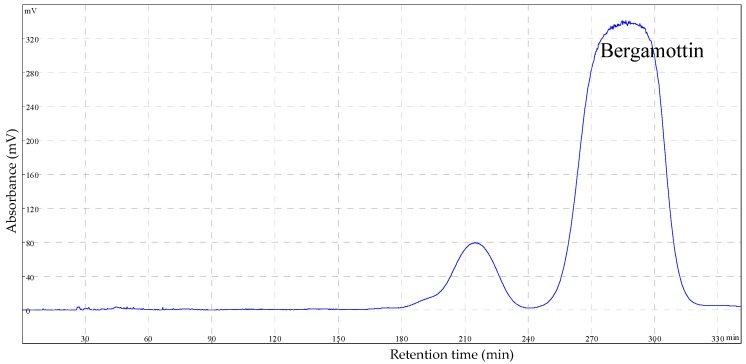
High-speed counter-current chromatography (HSCCC) chromatogram of the purification of bergamottin extracted from the silica gel-refined sample. Two-phase solvent system: hexane–ethyl acetate–methanol–water (1:1:2:0.625, *v*/*v*/*v*/*v*); stationary phase: upper phase; mobile phase: lower phase; flow rate: 2.0 mL·min^−1^; revolution speed: 900 rpm; detection wavelength: 250 nm.

In order to separate two similar substances simultaneously with HSCCC, it is important to choose a proper solvent system. A proper HSCCC system should have a partition coefficient (*K* value) range of 0.5–2.5 and a *K* ratio of about 1.5 or higher may result in good partition of two compounds with similar characteristics. It was reported that the chloroform–methanol–water system and the *n*-hexane–ethyl acetate–*n*-butanol–methanol–water system were efficient to isolate hydrophobic substances [26]. For example, the hexane–ethyl acetate–methanol–water (5:5:5.5:4.5, *v*/*v*/*v*/*v*) solvent system was used to separate four furanocoumarins from *Toddalia asiatica* (L.) Lam [27]. In our study, we tried eight different solvent systems based on literature and preliminary optimization experiments, among which hexane–ethyl acetate–methanol–water (1:1:2:0.625, *v*/*v*/*v*/*v*) resulted in *K*_1_ of 1.50 for the impurity, *K*_2_ of 2.19 for bergamottin, and the *K*_2_/*K*_1_ ratio of 1.46 (Table 2). As shown in the HSCCC chromatograph, such a solvent system resulted in the successful separation of bergamottin from the impurities (Figure 2). After the HSCCC experiment with the selected solvent system, 8 mL of the eluent was collected for each tube and tubes with pure bergamottin were combined for further analysis.

**Figure 3:**
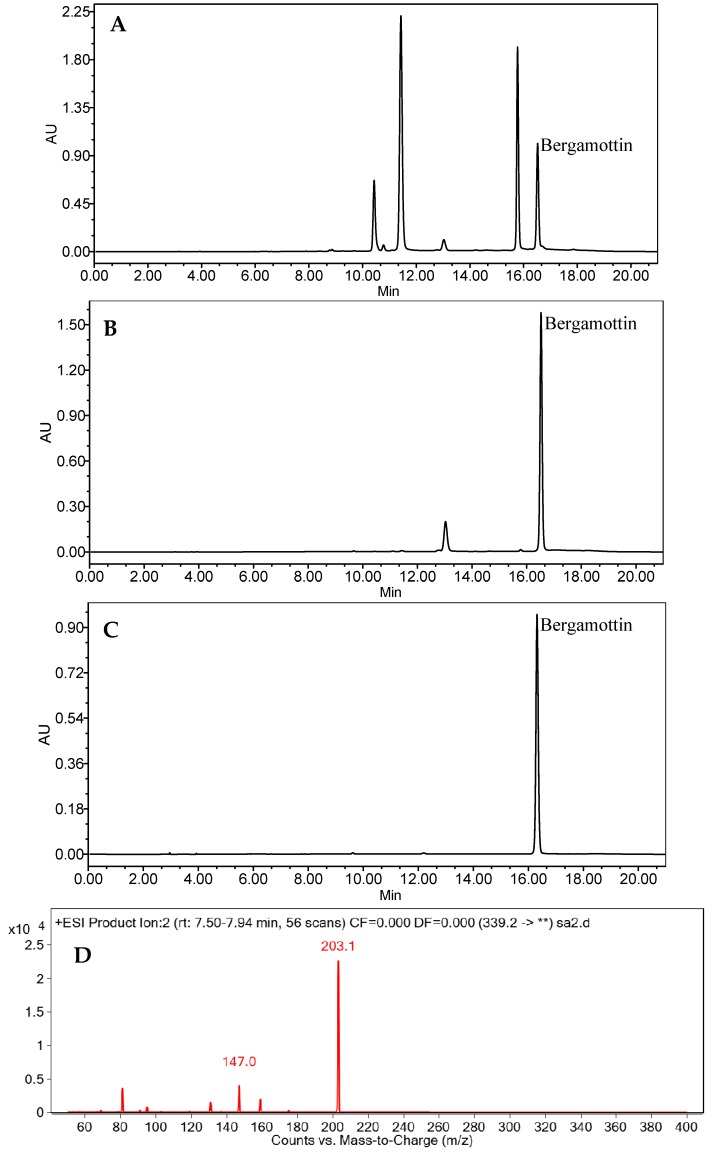
HPLC chromatogram of crude extract before (**A**) and after (**B**) treatment with silica gel column; the HSCCC purified product (**C**); LC-MS^2^ chromatogram of the final purified bergamottin (**D**) (λ = 250 nm).

HPLC analysis of bergamottin purification in different procedures was shown in Figure 3, and purities and recoveries of bergamottin in the two-step purification procedure were concluded in Table 3. The purity of bergamottin in crude extract was only 0.05%. After one-step silica gel column purification, many impurities were removed as shown in the HPLC chromatograms (Figure 3A,B), and the purity of bergamottin rose up to 44.82% in the silica gel-refined sample, which was nearly 900-fold that of the crude extract. The recovery of bergamottin in this step was 79.48% (Table 3). After HSCCC purification, 22.6 mg bergamottin with a purity of 94.01% was obtained and the recovery rate was 65.73% (Table 3; Figure 3C). The purified bergamottin was further identificated by LC-MS and nuclear magnetic resonance (NMR) spectroscopy. The [M + H]^+^ ion at *m*/*z* 339 suggested the molecular weight of bergamottin to be 338. In the LC-MS^2^ chromatogram, fragment ions of two typical products with *m*/*z* 203 [M + H − C_10_H_16_]^+^ and 147 [M + H − C_12_H_16_O_2_]^+^ were detected (Figure 3D), which was consistent with the previous study [28]. ^1^H- and ^13^C-NMR data for purified bergamottin were as follows: 

^1^H-NMR (400 MHz, CDCl_3_) δ 8.16 (1H, d, *J* = 9.8 Hz, H-4), 7.59 (1H, d, *J* = 2.4 Hz, H-9), 7.16 (1H, brs, H-8), 6.96 (1H, dd, *J* = 2.3, 0.8 Hz, H-10), 6.27 (1H, d, *J* = 9.8 Hz, H-3), 5.54 (1H, tq, *J* = 6.9, 5.8 Hz, H-2′), 5.14–5.01 (1H, m, H-6′), 4.95 (2H, d, *J* = 6.9 Hz, H-1′), 2.10 (4H, m, H-4′, 5′), 1.69 (3H, brs, H-9′), 1.68 (3H, brs, H-8′), 1.60 (3H, brs, H-10′).

^13^C-NMR (101 MHz, CDCl_3_) δ 161.46 (C, C-2), 158.29 (C, C-7), 152.83 (C, C-8a), 149.14 (C, C-5), 145.03 (CH, C-9), 143.21 (C, C-3′), 139.76 (CH, C-4), 132.20 (C, C-7′), 123.64 (CH, C-2′), 119.02 (CH, C-6′), 114.38 (C, C-6), 112.73 (CH, C-3), 107.70 (C, C-4a), 105.21 (CH, C-10), 94.40 (CH, C-8), 69.92 (CH_2_, C-1′), 39.64 (CH_2_, C-4′), 26.36 (CH_2_, C-5′), 25.82 (CH_3_, C-8′), 17.86 (CH_3_, C-10′), 16.82 (CH_3_, C-9′).

Therefore, all HPLC, LC-MS, ^1^H- and ^13^C-NMR data confirmed the purified compound as bergamottin.

### 2.3. Antiproliferative Activity of Bergamottin

In the present experiment, the antiproliferative activity of purified bergamottin was tested by using three cancer cell lines, i.e., HepG2, HL-60 and BGC-823 cells.

**Figure 4:**
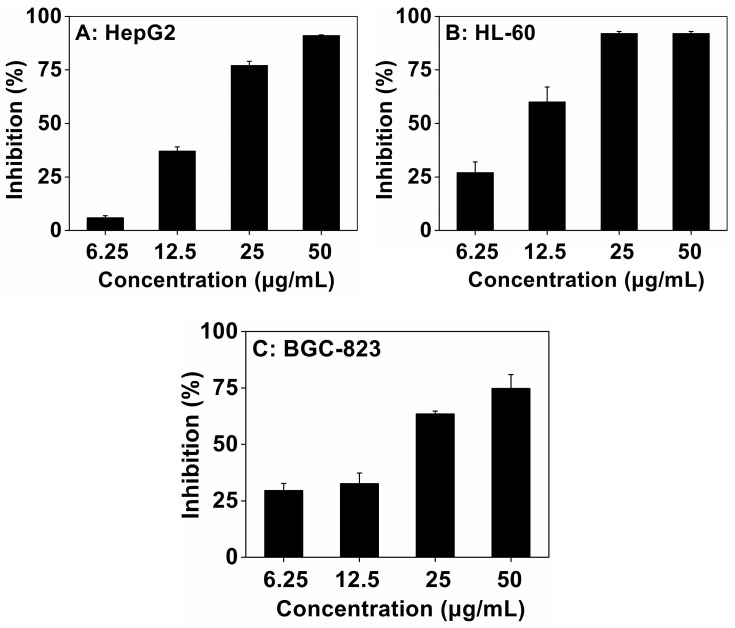
Effect of purified bergamottin on the growth of three cancer cell lines. Human liver cancer HepG2 (**A**); promyelocytic leukemia HL-60 (**B**); and gastric cancer BGC-823 (**C**) were used in the experiment. Taxol was used as a positive control.

From the results, it could be observed that bergamottin showed a significant inhibition effect on three cell lines, all in a dose-dependent manner (Figure 4). When the concentration of tested bergamottin increased from 6.25 to 50 μg·mL^−1^, the inhibitory rates on HepG2, HL-60 and BGC-823 increased from 6 to 91%, from 27 to 92%, and from 29.5 to 74.7%, respectively. Taxol was used as the positive control and the IC_50_ of taxol were 1.34 ± 0.36, 1.07 ± 0.11, and 252 ± 13 μg·mL^−1^ for HepG2, HL-60 and BGC-823 cells, respectively. Among the three cell lines, bergamottin exhibited the strongest inhibition effect on HL-60 cells, which resulted in an IC_50_ value of 8.63 μg·mL^−1^ and it was much lower than those of the other two cell lines (the IC_50_ values were 17.47 μg·mL^−1^ and 18.06 μg·mL^−1^ for HepG2 and BGC-823 cells, respectively). The high antiproliferative activity of bergamottin on the inhibition of the differentiation of HL-60 was reported previously [18]. Bergamottin at a concentration of 50 μmol·L^−1^ inhibited 50% invasion of human fibrosarcoma HT-1080 cells [1]. Also, bergamottin showed an inhibitory effect on human multiple myeloma U266 cells and lung cancer A549 cells in both time- and concentration-dependent manners [2,3]. However, it is the first report on the antiproliferative activity of bergamottin in the liver cancer and gastric cancer cell lines.

### 2.4. Glucose Consumption Activity of Bergamottin

**Figure 5:**
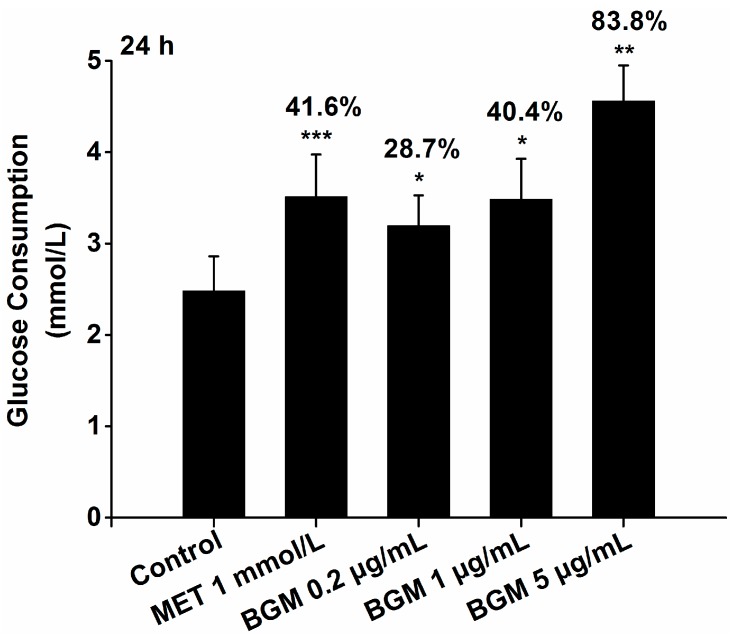
Effect of purified bergamottin (BGM) on glucose consumption after 24 h treatment in HepG2 cells. * *p* < 0.05, ** *p* < 0.01, *** *p* < 0.001, compared to the DMSO blank control. MET, metformin as a positive control.

Glucose consumption activity in HepG2 cells is an effective in vitro model for screening natural products with hypoglycemic potential. In our lab, it has been successfully used to discover the hypoglycemic activity of neohesperidin isolated from citrus fruit, which was confirmed by an in vivo experiment carried out in diabetic KK-A^y^ mice [21,29]. By using this experimental model, litchi fruit with high (−)-epicatechin content or high procyanidin content [30] and Chinese bayberry fruit with high anthocyanin and flaovonol content [31,32] were found to have great potential as hypoglycaemic food. In the present study, the glucose consumption activity of bergamottin was tested in HepG2 cells with 1 mmol·L^−1^ (i.e., 129 μg·mL^−1^) metformin (MET), a common oral hypoglycemic drug, as a positive control. Results showed that bergamottin significantly enhanced glucose consumption in HepG2 cells in a dose-dependent manner (Figure 5). Compared with the DMSO blank control, bergamottin remarkably increased the consumption of glucose in all detected concentrations, and at the concentration of 5 μg·mL^−1^, the glucose consumption in bergamottin-treated HepG2 cells increased by 83.8% (Figure 5). This is the first report on the potential antidiabetic effect of bergamottin. MET is reported to function in many ways, reducing the induction of hepatic glucose, decreasing glucose absorption, or improving insulin sensitivity, while the mechanism of bergamottin in the potential in vivo glucose regulation deserves further investigation.

## 3. Materials and Methods

### 3.1. Chemicals and Reagents

Bergamottin standard, methanol and acetonitrile of chromatographic grade for HPLC were bought from Sigma-Aldrich (St. Louis, MO, USA). Other analytical reagents were bought from Sinopharm Chemical Reagent (Shanghai, China). Silica gel was purchased from Qingdao Haiyang Chemical Co., Ltd. (Shandong, China). l-Glutamine, RPMI-1640 and Dulbecco’s modified Eagle’s medium (DMEM) were purchased from GIBCO (Grand Island, NY, USA). Cell Counting Kit-8 (CCK-8) was purchased from Dojindo (Kumamoto, Japan). Fetal bovine serum (FBS) was obtained from Hangzhou Sijiqing Biotec Co. (Zhejiang, China). Sulforhodamine B (SRB) was from Sigma-Aldrich. MET was purchased from Kunshan Double-Crane Pharmaceutical Co., Ltd. (Jiangsu, China). Glucose Assay Kit was bought from Nanjing Jiancheng Bioengineering Institute (Nanjing, China). Double-distilled water (ddH_2_O) was used in all experiments. All solutions for HPLC and LC-MS were filtered through 0.22 μm membrane before injection.

### 3.2. Fruit Materials

Mabuwendan, Yuhuanyou, and Sijiyou were collected in November of 2015 from Wenzhou city, Zhejiang province, China. Shatianyou was collected in November of 2015 from Nanning city, Guangxi province, China. YJZXY was collected in October of 2016 from Yongjia, Wenzhou city, Zhejiang province, China. White grapefruit was collected in November of 2015 from Quzhou city, Zhejiang province, China. Red grapefruit was bought in October of 2015 from Tmall fresh Supermarket of Shanghai, China. Mixiagan was harvested in December of 2015 from Xiangshan city, Zhejiang province, China. All fruits were divided into four parts, i.e., flavedo, albedo, SM and JS. Each tissue was ground into fine powder after lyophilization and stored at −80 °C for further analysis.

### 3.3. HPLC and LC-MS Analysis of Bergamottin

Bergamottin was analyzed according to Widmer and Haun [33] with some modifications. The HPLC system (Waters e2695, 2998 photo-diode array detector, Waters, Milford, PA, USA) equipped with a Sunfire^®^ C18 analytical column (4.6 mm × 250 mm, 5 μm) was used with Empower chromatography workstation. The mobile phase for HPLC consisted of ddH_2_O (A) and acetonitrile (B). The elution gradient was as follows: 0–1 min, 10% of B, 1–5 min, 10–80% of B, 5–10 min, 80% of B, 10–12 min, 80–95% of B, 12–15 min, 95% of B, 15–19 min, 95–10% B, 19–21 min, 10% of B. The flow rate was 1 mL·min^−1^ and the injection volume was 10 μL. Bergamottin was detected under 250 nm and primarily identified by the retention time and maximum absorption wavelength. A standard solution at concentrations of 31.25, 62.5, 125, 250, 500, and 1000 μg·mL^−1^ was prepared to quantify the content of bergamottin in samples.

Further identification of purified bergamottin was carried out by using the Agilent 6400 Triple Quadrupole LC-MS system (Agilent Technologies Inc., Santa Clara, CA, USA). It was operated in positive ionization and the specific operating conditions were as follows: capillary pressure 4000 V, atomizer 45 psi, dry gas velocity 5 L·min^−1^ at 325 °C. Data acquisition and processing was performed using Agilent MassHunter workstations. 

The ^1^H- and ^13^C-NMR data were obtained on a Bruker Avance 500 instrument (Bruker Biospin, Fallanden, Switzerland). Purified bergamottin (6 mg) was dissolved in 0.6 mL deuterated chloroform (CDCl_3_) in a 5 mm ф tube. Coupling constants (*J*) and δ (parts per million) were presented as chemical shifts.

### 3.4. Quantification of Bergamottin in Different Fruit Tissues of Different Citrus Cultivars

Freeze-dried powder (0.2 g each) of flavedo, albedo, SM and JS of each citrus cultivar was extracted with 4 mL of petroleum ether (60–90 °C) by sonication in an ultrasonic cleaner for 30 min. The mixture was centrifuged at 10,000 rpm for 10 min and the samples were extracted twice. Both extracts were combined and evaporated to remove solvents. The extract was then dissolved in 0.1–1 mL methanol of chromatographic grade for HPLC analysis.

### 3.5. Purification of Bergamottin from YJZXY Flavedo

#### 3.5.1. Preparation of the Crude Extract

The ground powder of YJZXY flavedo (80 g) was extracted with 1600 mL of petroleum ether by sonication for 30 min. The ultrasonic frequency was 60 kHz and power was 30 W. After filtration with filter paper (Whatman NO.1), the samples were extracted twice and both extracts were combined and evaporated with a rotary evaporator under vacuum at 30 °C to obtain the yellowish oily residue as crude extract. The residue dissolved in petroleum ether was used as crude extract for the subsequent experiments.

#### 3.5.2. Silica Gel Column Chromatography 

The obtained crude extract was dissolved in petroleum ether and then a proper amount of silica gel was added into the solution. After completely removing the petroleum ether under reduced pressure, the mixed powder for column chromatography was obtained and loaded onto the silica gel column (19/22, 46 mm × 457 mm) (crude extract:silica gel = 1:50, based on weight). The powder was then eluted with petroleum ether/ethyl acetate (13:1) solutions and 30 mL of the eluents was collected for each tube. Tubes with high bergamottin content were combined to obtain the silica gel-refined sample after removing the solvents.

#### 3.5.3. HSCCC Purification

A TBE 300A HSCCC (Tauto Biotechnique, Shanghai, China) equipped with three polytetrafluoroethylene coil separation columns (total volume, 315 mL; diameter of tube, 1.6 mm), a 20 mL sample loop, a TBP5002 pump, and a TBD2000 UV detector were employed in the present study. The temperature of the separation column was kept at 25 °C by a DC-0506 low constant temperature bath. Data were collected by a HW-2000 chromatography workstation.

The two-phase solvent system for HSCCC purification was selected by the partition coefficients (*K* value) of bergamottin. A proper amount of silica gel-refined sample was dissolved in the distinct pre-equilibrated solvent systems (*V*_upper phase_/*V*_lower phase_, 1:1) and then vortexed for mixture. After 3 minutes standing, each phase was analyzed for bergamottin by HPLC and the peak area was recorded as *A*1 and *A*2 respectively. Partition coefficient (*K*) = *A*1/*A*2.

The solvent was put into a separating funnel as the selected ratio and equilibrated to form two phases. Each phase was then separated and degassed in the ultrasonic cleaner for 30 min. The upper phase solvent was firstly pumped into the separation column of HSCCC at a flow rate of 30 mL·min^−1^. The apparatus was then rotated at 900 rpm and the lower phase was injected into the column at 2 mL·min^−1^. After reaching the equilibration, the retention rate of the stationary phase was calculated as 68% and the sample dissolved in the upper phase solvent was injected. The effluent was detected by a UV detector at 250 nm and 8 mL of it was collected for each tube for HPLC analysis. Tubes with pure bergamottin were combined to detect the purity and for further analysis.

### 3.6. Cell Lines and Cell Culture

HepG2 and HL-60 cells were bought from Shanghai Institute of Biology, Chinese Academy of Medical Sciences, and cultured in the College of Pharmaceutical Sciences, Zhejiang University. BGC-823 cells were purchased from JENNIO Biological Technology, Guangzhou, Guangdong, China. HepG2 cells were cultured in DMEM while HL-60 and BGC-823 cells were cultured in RPMI-1640. Each medium contained 10% heat-inactivated FBS, 2 mmol·L^−1^ l-glutamine (replaced with 20 mmol·L^−1^ hydroxyethyl piperazine ethanesulfonic acid for BGC-823), 100 μg·mL^−1^ streptomycin and 100 IU·mL^−1^ penicillin. Cells at log phase were used in the experiment.

### 3.7. Cell Proliferation Assay

Cells (5000 cells per well for HepG2, 6000 cells per well for HL-60, 8000 cells per well for BGC-823) were seeded in the 96-well microtiter plates and 200 μL medium was added into every well. After incubation overnight at 37 °C in 5% CO_2_, samples diluted to a gradient concentration with DMSO were added into the cells and then incubated for three days. To the control group was added DMSO.

Proliferation of HepG2 cells was detected by SRB assay [34]. After the treated cells were fixed with 10% trichloroacetic acid for 1 h, 70 μL SRB solution (4 mg·mL^−1^) was used to stain the cells for 20 min at room temperature, and then the SRB solution in cells was dissolved in 100 μL Tris-Base (10 mmol·L^−1^). The absorbance at 515 nm was read by a microplate reader (Thermo Electron Co., Vantaa, Finland). The inhibitory ratio of cell proliferation for every well was calculated as: inhibitory ratio (%) = (OD_control_ − OD_treatment_)/OD_control_ × 100%. The concentration inhibiting cell growth by 50% was recorded as IC_50_.

CCK-8 assay was used to evaluate the proliferation of HL-60 and BGC-823 cells according to Zhang and Sun [35] with modification. An amount of 20 μL CCK-8 solution was added to each well. After incubated for another 2 h, absorbance at 450 nm was measured by a microplate reader and the calculation formula of the inhibitory ratio was the same as that for the SRB assay. The IC_50_ value was also calculated.

### 3.8. Glucose Consumption Assay

HepG2 cells for glucose consumption assay were incubated in high-glucose DMEM with 10% FBS (37 °C, 5% CO_2_). The culture medium was changed every other day and passaged 2–3 days.

A glucose consumption assay was performed according to Li et al. [36] with some modification. Exponentially growing cells were seeded in 96-well microtiter plates with six wells left as blanks. After reaching 80 to 90% fusions, cells were washed with PBS twice. The medium was changed into serum-free RPMI-1640 with 0.2% BSA. MET was then added to the medium to obtain a final concentration of 1 mmol·L^−1^ or bergamottin serially diluted in DMSO. MET was the positive control and DMSO was the solvent control. After 24 h treatment, the glucose concentration of the medium was detected by the glucose oxidase method with the glucose assay kit. Briefly, 10 μL of medium in every well was mixed with 1000 μL test reagent from the kit. After being reacted for 20 min at 37 °C, the absorbance of the mixture was measured at 505 nm by the microplate reader. Glucose consumption was recorded as glucose concentrations of cell-free blank wells minus that of treated wells. In order to correct the experimental error caused by combined factors such as the number of cell inoculations and the cytotoxicity of compounds, cell viability was detected with SRB assay. Three independent experiments were carried out with three parallel wells in every experiment.

### 3.9. Statistic Analysis

Experiments were performed in triplicate and data are expressed as the mean ± standard deviation. Statistical analyses were performed with SPSS version 19.0 (IBM, Armonk, NY, USA). Student’s *t*-test was applied in Figure 5 and *p* < 0.05 was significant. Significant differences in Table 1 were calculated using one-way ANOVA, followed by Tukey’s multiple range test at *p* < 0.05.

## 4. Conclusions

Among different tissues of eight citrus cultivars tested in the present study, the flavedo of YJZXY was found to be a good source of bergamottin. Combination of silica gel column chromatography and HSCCC resulted in the effective removal of pigments in flavedo and purification of the low polar bergamottin. Purified bergamottin showed a significant inhibition effect on HepG2, HL-60 and BGC-823 cell lines, all in dose-dependent manners. In addition, bergamottin showed a significant increase in glucose consumption in HepG2 cells, which is the first report on its potential regulation activity in the glucose metabolism. Such a study will facilitate the comprehensive utilization of *Citrus* resources and provide technical support for further pharmacological studies on natural compounds such as bergamottin, which will lead to the development of the *Citrus* industry or functional products with various formats.

## Figures and Tables

**Table 1:** Bergamottin content in different tissues of eight citrus cultivars.

Cultivars	Bergamottin Content (μg·g^−1^ DW)			
	Flavedo	Albedo	SM ^1^	JS ^2^
*Citrus grandis*				
Mabuwendan	397.97 ± 3.02 ^c^	1.97 ± 0.08 ^c^	1.29 ± 0.03 ^ef^	13.98 ± 0.01 ^d^
Shatianyou	2.64 ± 0.09 ^f^	0.23 ± 0.00 ^e^	0.91 ± 0.01 ^f^	0.41 ± 0.00 ^e^
Sijiyou	356.04 ± 14.86 ^d^	0.91 ± 0.07 ^de^	3.46 ± 0.08 ^de^	34.07 ± 0.28 ^c^
Yuhuanyou	583.15 ± 12.42 ^b^	1.88 ± 0.02 ^cd^	3.70 ± 0.04 ^d^	57.57 ± 0.95 ^b^
Yongjiazaoxiangyou	666.54 ± 16.02 ^a^	15.87 ± 0.93 ^a^	7.23 ± 0.18 ^c^	1.23 ± 0.01 ^e^
*Citrus paradisi*				
White Grapefruit	11.43 ± 0.54 ^f^	0.44 ± 0.01 ^e^	0.91 ± 0.01 ^f^	0.84 ± 0.01 ^e^
Red Grapefruit	206.73 ± 3.95 ^e^	16.34 ± 0.05 ^a^	42.96 ± 2.23 ^a^	144.24 ± 3.00 ^a^
*Citrus reticulata*				
Mixiagan	212.53 ± 13.48 ^e^	12.58 ± 0.49 ^b^	12.06 ± 0.32 ^b^	0.57 ± 0.01 ^e^

^1^ SM: Segment membrane, ^2^ JS: Juice sacs. All data are presented as mean ± SD (*n* = 3) on a dry weight (DW) basis. Letters within each column followed by different superscript letters were significantly different (*p* < 0.05) according to Tukey’s tests.

**Table 2:** Partition coefficient (*K* value) of bergamottin in different solvent systems.

Solvent System (*v*/*v*/*v*/*v*)	Ratio	*K*_1_	*K*_2_^1^	*K*_2_*/K*_1_
Hexane–ethanol–acetonitrile–water	10:8:1:1	0.34	0.31	0.91
Chloroform–methanol–water	2:1:1	0.05	0.05	1
Chloroform–methanol–water	13:7:8	0.25	0.33	1.32
Chloroform–methanol–*n*-butanol–water	4:3:1:2	0.01	0.01	1
Hexane–ethyl acetate–methanol–water	1:1:2:0.625	1.50	2.19	1.46
Hexane–ethyl acetate–methanol–water	1:1:2:1	1.01	1.24	1.22
Hexane–ethyl acetate–methanol–water	2:1:2:1	2.80	5.91	2.11
Hexane–ethyl acetate–methanol–water	5:5:7:3	2.30	4.21	1.83

^1^ *K*_2_ represents *K* value for bergamottin.

**Table 3:** The purities and recoveries of bergamottin in the two-step purification procedure.

Purification Step	Purity (%)	Recovery (%)	Yield (mg)
Crude extract	0.05	/	/
Silica gel-refined sample	44.82	79.48	74.6 ^1^
HSCCC	94.01	65.73	22.6 ^2^

^1^ The amount of sample was obtained from 80 g raw material by the silica gel column. ^2^ The amount of compound was obtained from the 74.6 mg silica gel-refined sample after purity detection.
